# Healthcare professionals’ perspective on treatment burden and patient capacity in low-income rural populations: challenges and opportunities

**DOI:** 10.1186/s12875-021-01387-y

**Published:** 2021-03-09

**Authors:** Ruth Hardman, Stephen Begg, Evelien Spelten

**Affiliations:** 1 La Trobe University Rural Health School, 471 Benetook Avenue, Mildura, VIC 3500 Australia; 2 Sunraysia Community Health Services, 137 Thirteenth Street, Mildura, VIC 3500 Australia; 3 La Trobe Rural Health School, La Trobe University, PO Box 199, Bendigo, VIC 3552 Australia

**Keywords:** Treatment burden, Patient capacity, Healthcare providers, Qualitative research, Self management, Multimorbidity

## Abstract

**Background:**

The challenges of chronic disease self-management in multimorbidity are well-known. Shippee’s Cumulative Complexity Model provides useful insights on burden and capacity factors affecting healthcare engagement and outcomes. This model reflects patient experience, but healthcare providers are reported to have a limited understanding of these concepts. Understanding burden and capacity is important for clinicians, since they can influence these factors both positively and negatively. This study aimed to explore the perspectives of healthcare providers using burden and capacity frameworks previously used only in patient studies.

**Methods:**

Participants were twelve nursing and allied health providers providing chronic disease self-management support in low-income primary care settings. We used written vignettes, constructed from interviews with multimorbid patients at the same health centres, to explore how clinicians understood burden and capacity. Interviews were recorded and transcribed verbatim. Analysis was by the framework method, using Normalisation Process Theory to explore burden and the Theory of Patient Capacity to explore capacity.

**Results:**

The framework analysis categories fitted the data well. All participants clearly understood capacity and were highly conscious of social (e.g. income, family demands), and psychological (e.g. cognitive, mental health) factors, in influencing engagement with healthcare. Not all clinicians recognised the term ‘treatment burden’, but the concept that it represented was familiar, with participants relating it both to specific treatment demands and to healthcare system deficiencies. Financial resources, health literacy and mental health were considered to have the biggest impact on capacity. Interaction between these factors and health system barriers (leading to increased burden) was a common and challenging occurrence that clinicians struggled to deal with.

**Conclusions:**

The ability of health professionals to recognise burden and capacity has been questioned, but participants in this study displayed a level of understanding comparable to the patient literature. Many of the challenges identified were related to health system issues, which participants felt powerless to address. Despite their awareness of burden and capacity, health providers continued to operate within a single-disease model, likely to increase burden. These findings have implications for health system organisation, particularly the need for alternative models of care in multimorbidity.

**Supplementary Information:**

The online version contains supplementary material available at 10.1186/s12875-021-01387-y.

## Background

Lifestyle-related chronic diseases (CDs) such as diabetes, arthritis, cardiovascular and respiratory conditions require a long-term commitment to active self-management; however ongoing adherence is often poor. Known barriers to successful CD self-management include social, cognitive, biomedical and health system factors [1–5]. These factors frequently interact, leading to reduced adherence and CD escalation.

**Fig. 1:**
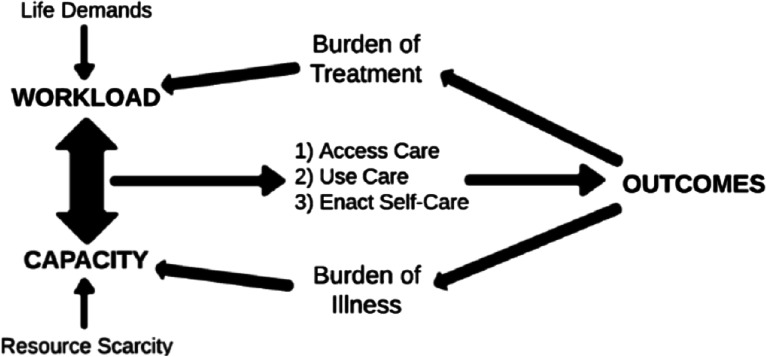
The Cumulative Complexity Model [6, 8]

Shippee’s Cumulative Complexity Model (CuCoM) [6] describes how different factors (such as poverty or polypharmacy) come together with the patient, their social environment and the healthcare environment to either promote or detract from a desired health outcome. In this model, complexity is not a medical diagnosis but a dynamic balance between patient workload (including self-management tasks, interactions with the healthcare system and everyday life demands) and capacity (including social support, socioeconomic resources and level of mental/physical functioning). The patient requires sufficient capacity to service their workload. Inadequate capacity or overwhelming workload may cause symptoms to escalate, which is then dealt with by intensifying treatment. Ironically, this increases workload even further and can result in a spiral of cumulative complexity [6–8], as illustrated in Fig. 1. The CuCoM is particularly applicable to people with multimorbidity (because of higher treatment workloads) and to those who are socially disadvantaged (since they have fewer resources), and can explain the poor outcomes and reduced adherence commonly seen in these groups [1, 4].

The concepts of workload and capacity have been explored in several qualitative studies [1, 9–11]. Although Shippee ‘s original concept of ‘workload’ refers to both direct treatment work and life demands, to increase clarity and consistency with the wider literature, we will hereafter use the term ‘treatment burden’ rather than ‘workload’. In line with other researchers, we define treatment burden as consisting of both direct treatment work and the impact on daily life, including work, social and caring responsibilities [12–14]. May [12], working with this definition, has proposed Normalisation Process Theory (NPT) [15, 16] as an appropriate tool to analyse treatment burden. This describes how new practices, such as learning how to manage chronic health conditions, become integrated into daily life, and has been successfully applied in several patient qualitative studies of treatment burden [13, 17]. Less attention has been paid to the concept of patient capacity as described in the CuCoM, although several taxonomies of capacity have been proposed [13, 18]. Boehmer [18] in a large qualitative review and synthesis used the acronym ‘BREWS’ to describe capacity as the interaction between **R**esource mobilisation, **W**ork realisation and **S**ocial functioning accomplished within a person’s **B**iographical reframing and **E**nvironment. This approach recognises that capacity is more comprehensive than a list of individual abilities or resources, and highlights its interactive, dynamic nature.

Although the Cumulative Complexity Model is supported by evidence from patient qualitative studies [1, 9, 11, 13], the concept is yet to be embraced by the healthcare system, as evidenced by reviews of medical records, clinical guidelines and CD management interventions [19–21]. Patients report that individual healthcare providers (HCPs) are often ignorant of burden and capacity factors [9, 10]; studies of HCPs support this view, noting a limited understanding of treatment burden [22], an ad hoc approach to the assessment of capacity [23], and discordant patient-practitioner perceptions of factors contributing to treatment burden [24].

Understanding burden and capacity is important for HCPs, not just because of their effect on treatment adherence, but because HCPs can directly influence these factors either negatively (by excessive treatment demands), or positively (by supporting capacity and reducing burden)[2]. This is even more applicable in vulnerable or disadvantaged populations who experience high levels of CD prevalence and multimorbidity, and whose life experiences may diverge significantly from the HCPs with whom they engage.

This study aims to explore how clinicians working in self-management support with rural socially disadvantaged populations understand and address burden and capacity factors in their patients. Short written vignettes describing patients were used to investigate HCP assessment and decision-making. Vignette responses have been shown to more closely approximate a clinician’s real-world behaviour than interviews, especially when looking at clinical decision-making, while also allowing motivators behind decisions to be explored in greater depth than in an observational study [25, 26]. The knowledge generated is intended to provide direction on ways of incorporating the concepts of workload, capacity and cumulative complexity into clinical practice, leading to improvements in treatment adherence and health outcomes.

We aimed to answer the following research questions:

1. Can HCPs working in chronic disease self-management support (CD-SMS) identify burden and capacity factors in patient case-studies (vignettes)?
2. How do HCPs working in CD-SMS understand burden and capacity, as described by Normalisation Process Theory (NPT) and the Theory of Patient Capacity (BREWS)?
3. What strategies do HCPs use to reduce burden or build capacity and what barriers do they identify?

## Methods

### Overall study design

This was a pragmatic qualitative study, analysed using the Framework Method [27]. We used the COREQ checklist for reporting of qualitative studies (see Additional file 1). Research was conducted in accordance with national ethics guidelines and approval was granted by the La Trobe University Human Research Ethics Committee.

### Participants and setting

All participants were clinicians (nurses and allied health professionals) working in chronic disease self-management support at two large (150–200 employees) regional community health centres in Victoria, Australia. SMS includes education, behaviour change interventions, goal-setting, symptom management and assisting with condition impacts on physical, psychological and social functioning [28]. In Australia, nurses and allied health professionals are the dominant providers of CD-SMS, both as first-contact providers and in collaboration with general practitioners (GPs). Community health centres cater for disadvantaged and low-income populations, many of whom experience complex multimorbidity. HCPs working in adult CD management at each health centre who described SMS as an integral part of their job were emailed with information about the study and invited to participate. Interviewees were purposively selected to ensure a range of different professions and years of experience. Data saturation was obtained after ten interviews, but a further two interviews were undertaken for confirmation.

### Interview process

Prior to commencing the interview, participants completed an informed consent form and a brief survey recording their demographic details. HCPs were then asked to read a vignette case study (described below) and to imagine that it was a referral for a new patient, presenting to them in their current role at the Community Health Centre. They were encouraged to verbalise any initial thoughts, using the ‘think aloud’ method [29], which reflects how clinicians typically respond when presented with a new patient. They were then asked to consider the vignette from two points of view—the patient, and the health provider – and reflect on the likely tasks that would need to be undertaken (burden) and skills required (capacity) for that person to successfully manage their health. Two vignettes (from a total of six case-studies) were selected for each clinician to view, chosen to closely reflect the HCP’s reported patient profile. Each vignette was commented on by four HCPs.

The second half of the interview consisted of general questions about the concepts of treatment burden, patient capacity and complexity, including the HCP’s thoughts about how such challenges could be overcome. Interview questions, including all vignettes, were trialled with two clinicians experienced in chronic disease management and modified in response to feedback. The interview protocol is available in Additional file 2.

### Vignette development/procedure

Six vignettes were constructed using interview data from thirteen multimorbid patients attending the same community health centres, who were part of a wider study. This approach enabled us to maximise data validity, by using case studies that closely represented the HCP’s usual patient population, whilst also addressing privacy concerns (since both patients and HCPs lived and worked in the same two rural communities) by blending and merging patient stories. Additional file 3 contains all six vignettes.

When writing a vignette, the use of both controlled variables, which provide the setting and context of the case-study but are not considered to greatly influence responses, and manipulated variables, which relate directly to the research questions, is recommended [25]. Table 1 describes each variable and their role in the vignettes. In this study, the controlled variables were age, gender and number and type of chronic conditions. Manipulated variables were of two types: information about environmental conditions (housing, family structure and source of income) and narrative features representing differing levels of patient capacity. The controlled and environmental variables were abstracted directly from the patient interviews and distributed across the six vignettes based on their frequency of occurrence in the patient interviews.

**Table 1:** Vignette design

	**Variables**	**Variables in each vignette () indicates number of vignettes which included the variable**
**Controlled variables**	Age	50–60 yrs (3 vignettes); 60–75 yrs (3)
	Gender	Male (3) Female (3)
	Chronic conditions	All vignette patients had at least 3 of the following conditions: musculoskeletal pain/arthritis (6); type 2 diabetes (4); diabetic sequelae (2); mental health (4); gut/bowel (3); cardiovascular (3); respiratory (2)
**Environmental variables**	Income source	Age pension (2); disability pension (1); unemployed (2); part-time work (1)
	Family situation	Living with spouse (3—all 60 + yr); spouse and children (1); single parent (1); alone (1)
	Housing	Rental (3); own home (2); mobile home (1)
**Narrative (capacity) variables**		**These factors were distributed across the vignettes**
	Physical	Diabetes complications, blood sugar control, multiple surgeries, functional or mobility impairments
	Personal	Mental health issues, motivation, memory
	Social	Family proximity, carer demands, quality of family relationships, family stressors (e.g. substance use), socially engaged or isolated
	Employment	Job loss, manual work history, self-employment, voluntary work, carer demands

The narrative features were based on four areas of patient capacity – physical, social, personal and employment – identified from the chronic disease management literature [3, 13, 18]. These features of patient capacity had previously been identified in the patient interviews and were distributed across the vignettes. Since this was a qualitative study it was not considered necessary to allocate variables using a factorial method; instead the aim was to provide a wide range of scenarios that closely represented the HCP’s daily caseload. All vignettes were written in the form recommended by Evans [25] to maximise realism and rigour.

### Analysis

Since our intention was to explore whether HCPs’ understanding was similar to or different from that of patients, we did not structure the interviews around the BREWS or NPT frameworks, instead asking general questions about burden and capacity. We wished to see whether HCPs were able to spontaneously identify burden and capacity domains (as described by BREWS and NPT) that had previously been identified from patient qualitative studies. After interview completion, we applied the same thematic constructs as in patient studies (BREWS and NPT) and tracked any data that did not fit this framework. We used the Framework Method for data analysis, working through each stage from familiarisation to interpretation [27]. Data was initially coded into the broad categories of burden and capacity. All data relating to burden was then coded to the four NPT themes of sense-making, relationship work, enacted work, and appraisal. All capacity data was coded to the five BREWS themes of biography, resource mobilisation, environment, work realisation, and social support. Table 2 describes key features of each burden (NPT) and capacity (BREWS) factor. All interviews were transcribed verbatim and initially coded by RH by hand. NVivo 12 software was then used and coding was reviewed and further explored by SB and ES. Disagreements were resolved in discussion with all three researchers.

**Table 2:** Burden and Capacity coding

**Normalisation Process Theory (NPT)**		**Patient capacity (BREWS)**	
Coherence (Sense-making)	Understanding the condition and treatments, planning care, setting goals	(B) Biography	Reframing to create a meaningful life that includes illness and treatment
Cognitive participation (Relationship work)	Obtaining support from family, friends and HCPs; managing difficulties in relationships	(R) Resource mobilisation	Access to, and ability to mobilise physical (energy, physical function); cognitive (literacy, memory); personal (resilience, self-efficacy); financial; and instrumental (time, transport etc.) resources
Collective action (Enacting work)	Carrying out work – adhering to treatments, making lifestyle and psychological adjustments, attending appointments	(E) Environment	Healthcare and social environments that fit with healthcare needs without interfering with other priorities
Reflexive monitoring (Appraisal)	Monitoring symptoms, reflecting on work undertaken and adjusting as necessary	(W) Work realisation	The experience of, and ability to normalise treatment workload as well as other life roles
		(S) Social functioning	Ability to socialise; practical social support; social acceptance of the patients’ CD and limitations; relations with HCPs

## Results

### Participant and interview characteristics

Twelve interviews were conducted with health professionals. Due to the COVID-19 pandemic, six interviews were via phone and six via video link, depending on interviewee preference and technology capacity. Eleven interviews were conducted by RH and one by SB. Six of the interviewees were known to RH who worked part-time as a clinician at one of the centres, but none of the participants were in a subordinate or supervisory relationship with RH. Interview duration ranged from 38 to 60 min (average 45′). Following interviews, brief field notes were made to record the key themes and impressions of the interview. All interviews were audio recorded and transcribed verbatim by RH. Table 3 records key characteristics of the health professionals and their reported patient profile.

**Table 3:** Characteristics of health professional interviewees

Location	Site 1: 7 participants, Site 2: 5 participants
Gender	All female
Age	24–56 years, mean 41 years
Profession	2 nurses; 4 diabetes educators (all nurses); 3 occupational therapists; 1 physiotherapist; 1 exercise physiologist; 1 podiatrist
Years since graduation	1–34 years, mean 13 years
Years in CDSM	1–18 years, mean 9 years
Specific postgraduate training in CDSM	7/12 reported formal training in CDSM
Reported typical patient population	Low socioeconomic status: blue-collar workers or healthcare card holders Age group: over 50 Chronic health conditions: Diabetes, COPD, cardiovascular disease, chronic pain, arthritis, anxiety/depression, obesity and multimorbidity

### Vignette validity

We addressed rigour and realism in the written vignettes by modelling the case studies on actual community health clients, trialling the vignettes with experienced clinicians and then presenting them to the participants in the form of a referral letter. During the interviews, we took further steps known to maximise validity [25] including matching the vignettes to each HCP’s reported patient population, asking the HCPs to respond as if the patient presented to them in their current role, and using a ‘think aloud’ process when responding to vignettes. We also asked participants to confirm that the vignettes were representative of their usual patients. This was strongly supported by the HCPs, who commented:

*”they are so typical… both of them” (B4) “it sounds like one of my clients…” (S3).*

### Ability of HCPs to identify burden and capacity factors in vignettes

HCPs were initially asked to ‘think aloud’ about each vignette, and then to consider barriers and enablers to CD management from both the patient and the HCP perspective. During both the ‘think-aloud’ and patient perspective responses, HCPs focussed on environmental stressors, especially life demands (work, caring), finances, social situation, and functional difficulties, rather than specific health conditions. When considering the vignette from the HCP perspective, the focus changed to treatment options, onward referrals and concerns about engagement with self-management. We compared the HCP ‘patient’ responses with the key capacity issues described in each vignette, based on the variables outlined in Table 1. This confirmed that all sociodemographic and capacity variables featured in Table 1 were identified and referred to by the participants and that the controlled variables were not unduly influencing responses. Table 4 illustrates the key issues in each written vignette and the participant responses. HCPs were easily able to identify the key issues in vignettes and often expanded on how these factors might impact on health management, especially in terms of the person’s ability to prioritise health in the face of other life demands, and their ability to access healthcare services to support them.

**Table 4:** HCPs’ responses to each vignette in relation to key capacity features

**Vignette no**	**Key capacity issues in vignette**	**Issues discussed by at least 3 HCPs** (each vignette was reviewed by 4 HCPs)
1 ‘Pete’	Complex multimorbidity and functional impairment, housing situation, limited family support	Likely high treatment demands, difficult housing situation, ability to access healthcare, reduced family support
2 ‘Angela’	Insulin dependent diabetic, some carer responsibilities, good social support	Low income, ability to prioritise health due to carer demands, good social support, needs good support for diabetes management
3 ‘Lyn’	Poor diabetes control, poverty, carer demands, lack of social support, family dysfunction, mental health	Inability to prioritise health due to life demands, mental health, social isolation, financial stress, needs significant support from healthcare system but access may be difficult
4 ‘Steve’	Work demands/stress related to business, long history of depression, poor diabetes management	Financial stress, depression, prioritising work over health leading to escalating health issues
5 ‘Mark’	Rural/isolated location, functional impairments, poverty	Functional limitations for day to day tasks, social isolation, ability to access healthcare, housing security, financial stress, health literacy
6 ‘Irene’	Caring responsibilities, social isolation, pain-related functional limitations, anxiety	Carer responsibilities affecting ability to prioritise health, social isolation, ability to access healthcare services

### HCPs’ understanding of burden and capacity, as described by the Theory of Patient Capacity (BREWS) and Normalisation Process Theory (NPT)

HCPs discussed capacity and burden specifically in relation to the vignette studies, but also more generally in terms of barriers and enablers, including ways to build capacity or reduce burden.

### Analysis of capacity

All HCPs were familiar with the concept of patient capacity and most reported undertaking a formal assessment of physical, social, economic and cognitive capacity for their patients. The Theory of Patient Capacity (BREWS) fitted the data well. Quotations related to a specific vignette have been noted (as V1,2 etc.)

HCPs discussed biography in terms of an individual’s future orientation. They discussed three possible responses for the vignette characters in managing their health. Firstly, denial and ignoring the future, associated with resistance to change and often (in the vignette portrayals) relating to the perception that immediate life demands were making it difficult for the person to prioritise their health.

*“… they haven't prioritised their own health for quite a while and they've just been working and putting food on the table … so sometimes there's some resistance to change … “(S3, V4)*

Secondly, viewing the future as an inevitable decline into old age and increasing disability.

*“…they just think they're getting older and this is just normal… we just put up with it…” (B5, V6).*

Finally, reframing which meant coming to terms with loss, seeing the future as positive and having meaningful goals.

*“…an acceptance of the situation and a hope for the future… understanding that you have this pain, the pain’s not going to go away but having hope that there [are] ways that you can manage it…” (S2, V5).*

HCPs considered that the ability to reframe identity and live a meaningful life with goals was vital for effective self-management. Some clinicians recognised that coming to this point could be very difficult since it meant dealing with loss and the realisation that life had changed permanently.

*“…it's not just for 6 or 8 weeks but for a lifetime and that's a lot to take on board…” (S1, V3)*

*“…there's sort of no quick fix for them there's no we'll fix it with this …it’s you have a chronic disease it's going to be there for the rest of your life…” (B4)*

#### Resource mobilisation

Resources fell into three categories: Physical, practical and personal. *Physical* resources related to illness burden and the functional impact on a wide range of daily activities, mood and sleep. HCPs identified chronic pain as the greatest contributor to illness burden, although other symptoms (fatigue and shortness of breath) were also discussed.

*“…he is probably noticing his back pain more than his erratic sugar levels…for people that have chronic pain it is often hard to see past the pain…” (B2, V4)*

*“…I would imagine (the pain) would have an effect on all the other things that are happening… so that would probably be where I would imagine Mark would want to …is get to the bottom of the pain…” (S6, V5).*

Practical resources included financial status, access to government or organisational support, and personal resources such as transport or computer literacy. Financial resources were considered by all interviewees to be one of the most significant barriers to capacity. Lack of money was particularly discussed in terms of its impact on treatment burden, affecting one’s ability to pay for appointments, medication, transport, healthy food and support services.

*“…financially he is on Newstart and he is rurally isolated … there is going to be the fuel cost plus the financial cost of paying the gap payment to see any specialists…” (S1, V5)*

*“…they are on the age pension they may or may not have money difficulties… transport or services…” (S2, V6)*

*“…she has been on the pension for the past 10 years…she'll probably be under some financial stress…” (S7, V2)*

Personal capacity included health literacy, cognitive abilities and mental health issues. HCPs rated health literacy (along with financial resources) as the most important contributor to capacity, but also saw it as closely connected to mental health, cognitive capacity and motivation.

*“… for some people, there are some huge health issues that have kind of never been explained to them properly by any health professional…” (S1)*

*“…[to] have the confidence to ask the right questions that I need to ask for my health… for example why am I taking that medication how is it going to help… if that doesn't work what is next what's my next step so having that confidence…” (S4).*

Potential mental health difficulties were discussed by most HCPs for every vignette, especially their interaction with physical symptoms, cognition and motivation.

*“…he's got a history of depression which is probably compounded now by all these other things…sometimes until that is dealt with they're not going to move forward with and they're not motivated to make the other changes…” (S3, V4).*

*“…when people have a lot of pain and then … that affects their mental health their ability to problem solve becomes quite impaired…” (S6, V5)*

#### Environment

Nearly all clinicians stressed the importance of a healthcare environment where a patient felt supported and listened to as important to build capacity, and saw the provision of this as an important part of their role. They also recognised that without this, patients often disengaged from healthcare.

*“… humans are about building relationships and that is in terms of your health relationships as well… you need to feel confident and comfortable with the healthcare professional that you are going to see…” (S5, V2).*

*“…trust and rapport… that really helps with self-management because they feel valued …that makes a big difference to the outcomes that the client has…” (S7)*

*“…I think a really big important one is the services that they have been engaged with in the past …if you've had a bad experience previously you are just likely to live with a bad health condition and not address it …” (S1, V5).*

The patient’s home environment was discussed both in terms of their housing suitability and security, and whether their life demands allowed people to prioritise their own health (most commonly referred to in relation to women with caring responsibilities).

*“…I think if she's got a lot going on in her life…it can be difficult to get people to worry about themselves when they are worrying about other people a lot…” (B1, V3)*

*“…he lives in a local caravan park which in my mind becomes relevant because of his living conditions… whether that is safe with his chronic back pain…” (B3, V1)*

*“…I would dare say that she probably puts other people's needs before her own and you know that will lead to a decline in her diabetes management …” (S5, V2)*

Stressful government-service environments such as the unemployment and child support systems were also referred to as factors that could impact on capacity.

*“…he’s on Newstart…that system is just going to set him up to drive that pain even further because of the stress that will put him under…” (S6, V5)*

#### Work Realisation

Many HCPs acknowledged the difficulty of successfully incorporating self-management work into daily life. Demands related to employment or caring were often associated with people not prioritising their health and thus reducing self-management ability. Most HCPs emphasised the importance of taking small steps and prioritising based on patient-identified goals and values. On the other hand, the successful achievement of treatment tasks was considered an important way to build capacity, by both increasing self-efficacy and reducing illness burden.

*“…at the next session say how did you go with that … I'll say you did do well maybe we can build on that … that increases their capacity to do things because they can see the benefit of what they've done …” (S3).*

#### Social Functioning

All HCPs referred to the importance of social networks and being connected to family, friends and community in terms of overall health outcomes, especially mental health. HCPs recognised that physical limitations and mental health interacted with social capacity.

*“…if we are talking about being socially isolated as well it's all that stuff drives people's mental health which will have an effect on his pain and vice versa…” (S6, V5)*

*“…how are those family connections and how does he feel about that… is he depressed or upset about that… is that going to affect his ability to look after his health…” (S5, V1)*

HCPs noted that social connections could increase access to resources (money, transport, home help) and enable the pursuit of meaningful activities, thus building biographical capacity.

### Analysis of burden

Apart from the diabetes educators, most HCPs were unfamiliar with the term ‘treatment burden’, but all presumed that it meant the demands of healthcare work. HCPs had a broad view of these demands and described both direct tasks such as pill-taking and attending appointments, but also life impacts such as the clash between treatment needs and family responsibilities, and the patients’ emotional burden of unremitting healthcare. Several also related it directly to patient capacity, describing how psychosocial stressors or resource deficits could lead to increased treatment burden. HCPs saw treatment burden as emerging both from specific treatment tasks and from difficulties in dealing with the healthcare system. This dual aspect of treatment burden has also been observed in patient studies [13].

#### Coherence

All HCPs considered that a patient’s understanding of their health condition(s) was vital for self-management and an important element of treatment work.

*“…I’d guess number one is finding out if Mark has any idea about pain… you really can't manage that until you get a good understanding of what the condition is…” (S6, V5)*

Participants had a broad conception of ‘Sense-making’. Making sense of health conditions was seen to be much more than learning a series of condition-specific skills or facts. It could enable people to take control of their health and plan a meaningful future. People’s beliefs, expectations and health literacy could make this task difficult. Some HCPs also acknowledged that the amount of knowledge required for effective self-management when there was co-morbidity could be overwhelming for patients.

*“…they don't have that knowledge so we have to provide that knowledge to them but then again it does become overwhelming the amount of knowledge that we are providing …” (S5)*

The literacy level of many educational resources, as well as differing and often inconsistent messages from different HCPs, was frequently identified as an issue.

*“…a lot of people just give out brochures and things like that and expect people to read them but they don't they just go in the bin…” (B1)*

*“…it can be overwhelming for people to be told lots of different things by lots of different health professionals who are looking after lots of different things…” (S1)*

#### Cognitive Participation

HCPs all stressed the importance of the patients engaging with multiple HCPs to manage their health. Each HCP recommended the involvement of at least three different HCPs per vignette, despite simultaneously recognising that this would increase the burden.

*“…people who are seeing multiple specialists …sometimes people are just ticking a box they are going to an appointment at times they are not sure why they're there and they are too overburdened to actually take anything on board…” (S1, V4).*

*“…the issue is what we all like to do is send people off to 7 different Professionals and then that can be …that's where we lose them sometimes isn't it so that's an issue” (S6)*

They also stressed the importance of the therapeutic alliance and their role as a facilitator working on mutually agreed goals, rather than a director of care. Many HCPs also recommended social services for the vignette patients (respite, home help, financial counselling) but noted that access was often limited.

Poor health service communication and co-ordination was acknowledged as a universal issue and a major contributor to burden. HCPs felt powerless to address these failings, which they believed could only be dealt with by more integrated technology and increased funding. Several HPs reported that these failings resulted in their own ‘treatment burden’ since they were often working outside of their roles to compensate for shortfalls in the system. This required time and emotional energy.

*“…it's not so much the number of clients that we are seeing in a day it's the level of…like there's an awful lot of emotional energy that goes into our work…” (B4)*

#### Collective action

HCPs listed a range of self-management tasks that the vignette patients would need to complete, including management of medication, appointments, blood sugar testing, diet, exercise, mental health and sticking to a routine. Integrating chronic disease management into daily life was recognised as potentially very time consuming especially for diabetics, those with caring responsibilities and those with multiple health conditions.

*“…things you can no longer do… you can no longer eat your time is not your own anymore because you have appointment after appointment after appointment at all different places … trying to keep up and manage your life around your health…” (B5).*

*“…lots of medications to take at home… things like exercise programs that people have to do at home …not being able to live the rest of your life because you’re always having to do things for your health…” (B1).*

Treatment costs, particularly specialist and psychology appointments, travel costs (given the rural setting) and the costs of home help or equipment were identified as burdensome. Services that were more affordable inevitably had long waiting lists or restricted eligibility. HCPs also described how patients often needed to attend multiple locations or appointments due to poor health service co-ordination. Improved service co-ordination, afterhours access and co-location were identified as factors that could assist patients to complete their treatment tasks.

*“…if you do a referral that's one thing but getting into that appointment or accessing the dietitian or the physio it's sometimes restricted and then they think oh what's the point I haven't got in so I won't bother…” (S3, V3).*

*“…some people just cannot afford the gap payments for psychologists…if you ask them to find $80 a fortnight some people just cannot afford that…” (B4)*

*“…not all services are in the same place and some services can change quickly depending on government funding…” (S4)*

#### Reflexive monitoring

HCPs referred to this in the vignettes when discussing patient priorities and the need for the patient to decide what was important to them in terms of their health management.

*“…for Mark it's a case of … getting him to prioritise what would he like to achieve in life and then what would it take to get where he wants to be so what steps could we put in place…” (S1, V5).*

They recognised that many people would not be able to achieve all treatment tasks and that it was appropriate to reflect on and plan for what was possible rather than ideal.

*“…you're not trying to solve all of their health issues …just if you can make one thing easier for them today sometimes that's a really important thing… and I think that is often missed… there [are] constant demands that the patient achieves everything all of the time and it is unrealistic … that if they can achieve something they should be really proud of that…” (B3).*

### Strategies to reduce burden and increase capacity, and barriers identified.

#### Building capacity

HCPs reported that the combination of insufficient income, excessive life demands and poor mental health often impacted capacity cumulatively.

*“…the psychosocial stuff in the background that makes it complex …the finance, the family situation, the culture, the language… all those additional things that are outside of the biomedical situation …” (S7).*

*“…they have numerous health conditions or a range of health conditions… their home situation they might have a complex family or socioeconomic status whether there is a range of barriers…” (B2)*

They considered that accepting, understanding and being confident in treatment management was key to increasing capacity, although most felt that patients would find it difficult to do this on their own and would need ongoing support from a HCP, as well as available time and the right ‘head space’ to achieve this.

*“…a lot of it does come down to relationships with our clients and linking them into services that can help… link them in and sticking to what's important to a person…” (S2)*

HCPs considered that health literacy (which included both understanding and accepting chronic health conditions) and financial resources were the most important factors influencing capacity, closely followed by mental health status. Participants felt that they could assist in building capacity by improving health literacy, providing symptom management strategies and creating a supportive environment, but they often felt powerless to address issues related to finances, life demands and mental health.

*“…finances, finances, finances and finances… I think the vast majority of people that we see are surviving very few are thriving…” (S1)*

*“…oh god we just need more money… people with complex care needs need to be able to access things without having to pay a gap…” (B3)*

*“…one of the biggest challenges when their mental health is a long-standing mental health issue that has never been adequately addressed … sometimes we are seeing people and it has been 40 years…when that's been something that has driven a lot of their health concerns the whole time and trying to unpack that 40 years later is challenging…” (S6).*

*“…I think someone's mental health is going to be one of the most important things… if they're mentally not in a space that they feel that they can change or where they feel they are not in control then I think you're fighting a battle that is out of your control…” (S7).*

### Reducing Burden

Interviewees thought that a patient’s ability to reduce burden independently of HCPs or the health system was quite limited, apart from prioritising and routinizing self-care tasks where possible. They noted that capacity-building strategies (as listed in Table 5) could also assist with perceived treatment burden. HCPs struggled with many health system barriers which increased treatment burden but could not be easily addressed either by the patient or the individual HCP. Lack of adequate and consistent funding for services, and service co-ordination were identified as the biggest factors contributing to treatment burden.

**Table 5:** HCPs views: Factors that reduce burden or build capacity

**Reducing burden**	• Assistance with system navigation • Knowledge of available resources and greater access (waitlists, funding for equipment and social services) • Improved access to specialists and mental health services (telehealth, transport support, no gap payments, address waitlists and workforce). • Technology to improve service co-ordination (shared healthcare information plus time to read it) • Supportive HCPs who are patient-centred • Sustainable (long-term) service funding
**Increasing capacity**	• Available income • Understanding their condition and the point of treatment, being confident in management • Acceptance of condition and recognition of the need to address it • Ability to prioritise health • Living in a healthy environment • Availability of services (home help, respite) • Having goals and a purpose • Early provision of services (before people become too disabled) • Good mental health • Good social relationships
**Both**	• Established routine/integrating treatment into life, able to troubleshoot and prioritise • Manageable life demands (e.g. caring role)

*“…where there are multiple services if they are all in one place it helps to co-ordinate your care…” (S4)*

*“…there is a lot of jumping to and fro between various organisations as well…” (B5)*

*“…we have some clients who aren't eligible with home care packages but it really would be beneficial for them…so I think funding has a lot to do with it…” (B2)*

HCPs frequently suggested ways to reduce treatment burden, then immediately discounted them as being unrealistic.

*“…it would be really nice if we had multiple access to multiple providers in one location that they could get into at one time…that would be nice… it's a bit pie in the sky…” (B3)*

Many saw telehealth as a positive development to reduce costs and increase access, but there were concerns about computer literacy and broadband access in low income populations. Disengaging from healthcare altogether was noted to be one way that patients might deal with a high treatment burden. The issue of multiple appointments with different people, each focussing on a different part of the body, was recognised as a challenge that could not be easily solved, especially due to the sheer number of treatment options available.

*“…it's lovely that we have so many services but that just adds to the [feeling of] being overwhelmed really doesn't it…” (S6)*

Several participants noted that many patients did not have a strong relationship with their general practitioner (GP). This was a common issue in rural areas due to workforce shortages and transient staffing and reduced the likelihood of co-ordinated care.

*“…when I have had someone who has come in and they are complex it's not often that the general practitioner is all over it…I think that the GP can become overwhelmed in that scenario…” (S5)*

These factors are summarised in Table 5.

## Discussion

### Main findings

This study aimed to investigate how HCPs working in CD-SMS understood the elements of complexity, as described by the Cumulative Complexity Model. Our use of structured vignettes, rather than patient histories, allowed us to concentrate on specific capacity variables without compromising anonymity. All study participants were able to identify and discuss burden and capacity factors in the vignettes, and the data could be analysed using Normalisation Process Theory (NPT) and the Theory of Patient Capacity (BREWS), previously only explored with patients. HCPs listed a wide range of strategies to reduce burden or build capacity, but frequently reported health system challenges in implementing these strategies. HCP views were consistent across a wide range of disciplines and years of experience, although experienced clinicians were more likely to highlight the interaction between motivation for self-management and contextual factors such as low education.

### HCPs views compared with the literature

#### Patient literature

The study findings are strongly consistent with literature exploring the taxonomy of burden and capacity [11, 13, 18], with the HCP accounts describing all burden and capacity components. The interacting nature of burden and capacity [9, 13, 30, 31], especially how increased capacity can reduce burden, was also discussed by the HCPs. The HCPs also characterised burden as comprising both treatment tasks and health system deficiencies, as described by Gallacher et al. [13].

#### Health provider literature

The ability of HCPs to recognise patient burden and capacity constraints has been questioned in several studies [9, 10, 22]. HCPs are reported to focus on biomedical [24, 32] or motivational [23] rather than social-contextual factors when assessing treatment burden or capacity to self-manage. The current study offered a different perspective which may be related to the setting.

Study participants were all HCPs working with rural populations in community health settings, where there is an explicit commitment to the social model of health. In this setting, HCPs were highly cognisant of burden and capacity issues, and their comments on the vignettes were comparable to the patient literature. Other studies have interviewed GPs (physicians) and practice nurses in primary care, where there may be less understanding of SMS [33, 34] and limited access to interdisciplinary services. In contrast, community health CD services are often structured around the Chronic Care Model [35] and HCPs working in this environment generally have more time, greater expertise in SMS (with 7/12 interviewees reporting formal postgraduate training in this area) and access to interdisciplinary services.

Several studies have recommended that HCPs become more aware of access, resource and treatment burden factors in individual patients and tailor treatment accordingly [10, 23, 30], including an increased focus on patient-identified values, preferences and non-medical goals [10, 18, 24, 36, 37]. In the current study, such approaches appeared to be well-established. Even if participants were unfamiliar with the specific term ‘treatment burden’, they all recognised the importance of avoiding overwhelming treatment demands. Formally identifying and prioritising burden and capacity factors using available tools and measures [38, 39] could provide additional assistance to patients and HCPs, but many burden-capacity challenges require system-level changes that are out of reach of the individual patient or health provider. This echoes findings in a review of integrated care for multimorbidity [40], which noted that successful implementation needs macro-level change, but that most interventions occur at the micro- or meso- level.

### Recommendations and challenges

All HCPs felt that their efforts to assist with burden and capacity were limited by contextual factors over which they had little power. Consistent with other literature [41, 42], some experienced their own personal ‘treatment burden’ in trying to fill the gaps of poor service provision, and others stepped outside of their role to provide additional support or co-ordination if it was unavailable in their healthcare setting. They felt that many barriers could only be dealt with by the injection of more money and practical approaches including better technology, administrative support, stronger linkages between health and social services and time allocated for HCPs to communicate directly to each other.

HCPs also recognised that some of their own actions could increase treatment burden, for example referring the patient to multiple services, most of which were not ‘joined-up’. Even with increased funding, the single disease model of healthcare inevitably leads to patients being reduced to body components, with each piece needing treatment by a different person. Without care co-ordination, this results in excessive burden or disengagement. Several HCPs described the care co-ordination role as an ‘extra’ job they often assumed to help the patient, but to be effective this role needs to be both remunerated and formally recognised by the patient and all other HCPs working with that patient, especially the GP.

Dealing with mental health issues within the context of multimorbidity was seen as particularly challenging. Often the only response was to send the patient off to yet another service provider, this time to deal with their ‘head’. Despite the prevalence of co-occurring mental and physical health conditions [43], HCPs and health services continue to work within single-disease models [33, 40, 44] and alternative approaches are needed to avoid burden-capacity imbalance. One suggested approach is for HCPs to use and promote treatments that are effective in a range of conditions (e.g. exercise) so that the same intervention can address multiple health conditions [44]. Greater emphasis on generalist skills that reflect common comorbidities may also help to support patients who cannot manage yet another referral. Given the two-way relationship between anxiety, depression and many chronic health conditions, skills such as capacity coaching, trauma-informed care and mental health first aid [45–47] are likely to be particularly useful.

### Strengths and limitations

A strength of this study is the vignette methodology, which enabled us to explore how HCPs might actually respond to patients rather than being reliant on their explicit or theoretical knowledge. By using NPT and the theory of patient capacity, we could compare the data to the wider patient literature, strengthening the validity of the research. Interviewing HCPs who directly provide SMS, rather than GPs in primary care for whom SMS is a secondary role [48], resulted in a different perspective: one informed by negotiating the practical details and challenges of self-management. Requesting HCPs to imagine the patient’s perspective when viewing the vignettes (which is known to affect empathy) may have contributed to increased recognition of burden and capacity; however, these factors were also identified at the initial ‘think aloud’ stage.

The study findings are limited by the fact that the setting is a low income rural population with HCPs who work within a specific model of healthcare and may therefore be more aware of social-contextual issues. Despite this, the community health environment is valuable to explore because there are likely to be greater numbers of people with psychosocial complexity and multimorbidity. Such individuals are at greater risk of burden/capacity imbalance and disengagement from the healthcare system than more advantaged populations. Additionally, in this setting many of the recommendations to address burden and capacity (such as increased HCP awareness and tailored care based on patient priorities) have already been addressed, yet significant challenges remain.

Although challenges related to healthcare costs and accessibility are more relevant in settings with resource and workforce shortages, system co-ordination issues are widespread across all health systems [31]. Therefore, it is likely that the study findings are relevant in other settings.

## Conclusions

HCPs in community health settings have a good understanding of burden and capacity, and the impact of these factors on the ability of their patients to self-manage chronic health conditions. Many of the barriers to address burden and capacity are at the health system or societal level and are difficult to address. Despite their understanding of burden and capacity constraints, HCPs still operate according to a single disease model which may lead to increased burden. More systematic approaches to support patients (e.g. care co-ordination) and/or alternative care models for multimorbidity are needed to support patients in their chronic condition self-management.

## Supplementary Information

**Additional file 1.** COREQ checklist.

**Additional file 2.** Interview protocol.

**Additional file 3.** Vignette case-studies.

## Data Availability

The datasets used during the current study are available from the corresponding author on reasonable request.

## Abbreviations

CD: Chronic disease
CuCoM: Cumulative complexity model
NPT: Normalisation process theory
BREWS: Theory of patient capacity
HCP: Healthcare provider
SMS: Self-management support
