# Supplementary material for: Healthcare professionals’ perspective on treatment burden and patient capacity in low-income rural populations: challenges and opportunities
Source: BMC Fam Pract. 2021 Mar 9;22:50. doi: 10.1186/s12875-021-01387-y (PMC7942213; doi:10.1186/s12875-021-01387-y)
Supplement: Supplementary file 2 — Additional file 2. Interview protocol. [file 12875_2021_1387_MOESM2_ESM.docx]

**Interview protocol Burden and Capacity: Healthcare Providers**

**Prior to commencement of interview, review the participant information and consent form, reiterate information and ensure participant understands by asking them to state their understanding of the study and procedures.**

**Introduction:**

In this research we are interested in finding out more about how health professionals assist people to manage their chronic health conditions. We have previously collected information from people with chronic health conditions about the challenges they experience with self-care. We are aiming to discover whether health professionals and clients have the same or different perceptions of these challenges and how to best deal with them.

We hope that this research will enable us to identify ways of better supporting both health providers and people dealing with chronic health conditions. Everything you say in this session will be confidential and no identifying features (such as your name) will be used in this research. If you wish, a printed transcript of the interview can be sent to you. If at any time you want to stop, or have a break, please feel free to let me know.

**Part 1: Vignette case studies**

For the first part of this interview I would like you to read and discuss two case studies. Remember, there are no right or wrong answers – we are just interested to see how health professionals assess and make decisions about their clients.

(show first vignette)

Take a few minutes to read the first study. Imagine that it describes a client who is presenting to you for the first time. When you are ready, could you verbalise the initial thoughts that come into your head when reading about this client – the key issues that first strike you.

**Now** I want you to try and think about the case-study in two ways – first from the perspective of the person in the story; and then from your perspective as their health provider.

1. What would X need to do to manage their health successfully - what tasks do you think they would need to do?

‘Tasks’ should be understood in a broad sense: covering anything a person might need to do (including thought processes and practical activities) to manage their health.

1. What difficulties might X encounter and what resources or abilities might they need to help?

(Prompts: think about strengths they might have as well as challenges)

Now thinking from your perspective as their health provider:

1. In your current role, how might you assist X with their health management?

(Prompts: Providing education? Referrals? Specific treatments? Emotional support?)

1. What challenges might you as a healthcare worker face in providing assistance to X? How might these things be overcome?

(Prompts: Dealing with multiple health issues? Lack of time? Setting goals/prioritising? Factors outside your control?)

Repeat process for the second case-study.

**Part 2: General interview questions**

We would now like to explore more generally how you assess people who come to see you and how you make decisions about the best ways of assisting them in self-management.

Firstly, thinking about treatment burden

1. What does the term ‘treatment burden’ mean to you?
2. What things might ‘treatment burden’ include?
3. How would you decide if a person has a high treatment burden or not?
4. How can treatment burden be reduced? Consider this in 3 ways:
   - How an individual (person with a chronic disease) could reduce their burden?
   - What can health providers do to reduce burden?
   - What can health organisations, communities or the wider healthcare/political system do to reduce burden?

Now thinking about individual capacity:

1. How do you determine whether a client has sufficient capacity to manage their health?
2. Which factors do you think are most important?
3. What do you consider the commonest barriers or challenges to client capacity?
4. How can capacity be increased? Again, think about this in 3 ways:
   - What can the individual client do?
   - What can health providers do?
   - What can health organisations, local communities or the wider healthcare/political system do?

Finally, I would like your thoughts on client complexity:

1. How do you identify or classify a client as ‘complex’?
2. What factors are most important in classifying them as complex?
3. When dealing with a ‘complex’ client, what things might you need to do differently?

(Prompts: types of treatment you offer? appointment structure/time? additional supports or referrals?)

1. As a health provider, what do you find most challenging when dealing with complex clients?

This is the end of the interview:

Except to ask:

Did you feel that the case studies were a realistic representation of clients you might see in your clinical role?

Is there anything else you wish to say on this topic that you don’t think has been covered?

Thank the participant and reiterate that all they have discussed is confidential.
