# Supplementary material for: Healthcare professionals’ perspective on treatment burden and patient capacity in low-income rural populations: challenges and opportunities
Source: BMC Fam Pract. 2021 Mar 9;22:50. doi: 10.1186/s12875-021-01387-y (PMC7942213; doi:10.1186/s12875-021-01387-y)
Supplement: Supplementary file 3 — Additional file 3. Vignette case-studies. [file 12875_2021_1387_MOESM3_ESM.docx]

**Vignette case-studies**

**Vignette 1**

**Pete** is 70 years of age and lives with his wife Jan, aged 68. They are on the age pension and live in a transportable home which they own, in a local caravan park. Pete has had chronic back pain for 20 years, with several surgeries. He was diagnosed with COPD 3 years ago, at which time he stopped smoking. He has had type 2 diabetes (NIDDM) for 20 years with few problems, but he has developed diabetic foot ulcers over the past 3 years which prevent him from walking far. Pete and Jan are on their second marriage and have no contact with Jan’s children and grandchildren due to a relationship breakdown, but have established some good friendships at the caravan park.

**Vignette 2**

**Angela** is 52 and lives in secure rental accommodation with her two of her children (aged 19 and 21), one of whom has a learning disability. Both children are working, one in a supported environment. She has been on the disability pension for the past 10 years due to type 2 diabetes (insulin-dependent for 3 years), reflux and chronic shoulder pain related to an injury sustained when working as a personal carer. She does voluntary work a few times a week and has a close circle of friends and family. Her elderly parents live nearby and have some health issues, but are physically independent.

**Vignette 3**

**Lyn** is 54 and lives in rental accommodation with her partner Bob who is currently employed as a truck driver. Their granddaughter Anna (aged 6) lives with them 3 days a week; Lyn and Bob’s only child Teresa, who lives locally, has a long history of substance abuse and Anna has been under a shared custody arrangement since birth. Lyn has been unemployed for 5 years and receives the newstart payment; she previously worked in a supermarket. Lyn has worsening chronic neck and back pain and was diagnosed with type 2 diabetes (non-insulin dependent) and hypertension 3 years ago. She has had depression for the past 10 years. She has poor glycaemic control and is inconsistent in blood sugar testing. She has early signs of diabetic retinopathy. Bob is a supportive partner and tries to help Lyn in daily tasks wherever possible, but is often away due to work.

**Vignette 4**

**Steve** is 63 years old and lives with his wife Sue. They live in a small unit, which they own, and have together run their own cleaning business for many years. Steve has suffered from depression for 20 years. He has been overweight for years and was diagnosed with type 2 diabetes (NIDDM) 5 years ago. He has erratic blood sugar levels, and also experiences chronic back pain and irritable bowel syndrome. Because of back pain, Steve has had to reduce his working hours and they are struggling to keep the business going. Steve and Sue have 3 children, two of whom live locally, and several grandchildren, but they find it hard to see them regularly due to the demands of their business.

**Vignette 5**

**Mark** has chronic back, knee and shoulder pain and walks with a stick. He underwent a coronary bypass 3 years ago. He is finding it difficult to manage tasks around the house and garden. He is 57 years old and has lived alone, in a rental property some distance outside town, since his marriage break-up 10 years ago. He has been unemployed for the past 4 years and receives the newstart payment; previously he worked as a farm labourer but was unable to manage the physical demands. He struggles to leave the house and has become socially isolated, although he has a circle of mates who keep in touch and his daughter and young grandchild do visit regularly.

**Vignette 6**

**Irene** is 66 years old and lives in her own home with her husband Dave (68). They receive the age pension; Dave worked in warehousing but had to stop work aged 60 due to worsening cardiovascular health. Irene has been his carer since that time. Irene is overweight and has widespread osteoarthritis, asthma and reflux. She is finding household tasks more difficult to complete, reporting fatigue and pain after walking or standing for more than 10 minutes. She has had anxiety for many years. Irene and Dave moved to the country when they retired and have not developed many local friendships. Their two adult children are in regular communication, but both live interstate.
